# Role of miR-424 in the carcinogenesis

**DOI:** 10.1007/s12094-023-03209-2

**Published:** 2023-05-13

**Authors:** Soudeh Ghafouri-Fard, Arian Askari, Bashdar Mahmud Hussen, Mohammad Taheri, Nader Akbari Dilmaghani

**Affiliations:** 1 Department of Medical Genetics, Shahid Beheshti University of Medical Sciences, Tehran, Iran; 2 Phytochemistry Research Center, Shahid Beheshti University of Medical Sciences, Tehran, Iran; 3 Department of Clinical Analysis, College of Pharmacy, Hawler Medical University, Kurdistan Region, Erbil, Iraq; 4 Institute of Human Genetics, Jena University Hospital, Jena, Germany; 5 Urology and Nephrology Research Center, Shahid Beheshti University of Medical Sciences, Tehran, Iran; 6 Skull Base Research Center, Loghman Hakim Hospital, Shahid Beheshti University of Medical Sciences, Tehran, Iran

**Keywords:** miR-424, Cancer, Biomarker

## Abstract

Recent studies have revealed the impact of microRNAs (miRNAs) in the carcinogenic process. miR-424 is a miRNA whose role in this process is being to be identified. Experiments in the ovarian cancer, cervical cancer, hepatocellular carcinoma, neuroblastoma, breast cancer, osteosarcoma, intrahepatic cholangiocarcinoma, prostate cancer, endometrial cancer, non-small cell lung cancer, hemangioma and gastric cancer have reported down-regulation of miR-424. On the other hand, this miRNA has been found to be up-regulated in melanoma, laryngeal and esophageal squamous cell carcinomas, glioma, multiple myeloma and thyroid cancer. Expression of this miRNA is regulated by methylation status of its promoter. Besides, LINC00641, CCAT2, PVT1, LIN00657, LINC00511 and NNT-AS1 are among lncRNAs that act as molecular sponges for miR-424, thus regulating its expression. Moreover, several members of SNHG family of lncRNAs have been found to regulate expression of miR-424. This miRNA is also involved in the regulation of E2F transcription factors. The current review aims at summarization of the role of miR-424 in the process of cancer evolution and its impact on clinical outcome of patients in order to find appropriate markers for malignancies.

## Introduction

MicroRNAs (miRNAs) are transcripts with sizes around 23 nt that can influence expression of genes at post-transcriptional level. These small, highly conserved transcripts are transcribed by RNA polymerases II and III. The miRNA precursors generated by these polymerases go through a group of cleavage actions to make mature miRNAs [1]. miRNA precursors are lengthy polycistronic RNAs that share some features with mRNAs since they possess distinctive 5′ and 3′ borders, 7-methyl guanylate caps and poly(A) tails.

The function of miRNAs in the regulation of genes expression is accomplished via the RNA-induced silencing complex (RISC) [2]. After assemblage into RISC, miRNAs activate this complex to target mRNAs in a specific manner [1]. miRNAs which control oncogenes and tumor suppressor genes, are differentially expressed in various human malignancies and play a central role in all cancer hallmarks, especially in their real targets [3–6]. Moreover, miRNAs differ in their transcriptional units and the mechanisms of their regulation within genomic loci. Those being located within an intron of a host gene are transcribed in the identical orientation with primary transcript by the promoter of the host gene [7]. On the other hand, miRNAs located in the intergenic loci have their own promoters [7, 8].

miR-424 is encoded by a gene located on chr Xq26.3. This miRNA has been demonstrated to be dysregulated in different cancers. Notably, dysregulation of miR-424 in cancer samples have been associated with invasive behavior of malignant cells. However, different studies have reported various results regarding its expression in different cancers. Mechanistically, several lncRNAs act as molecular sponge for miR-424 to regulate its expression. The current review aims at summarization of the role of miR-424 in the process of cancer evolution and its impact on clinical outcome of patients in order to find appropriate markers for malignancies.

## Cancer cell lines

An experiment in neuroblastoma cell lines has revealed down-regulation of miR-424 and up-regulation of its target gene DCLK1 in these cells compared with normal spongiocyte cells. Mechanistical studies has confirmed that the role of miR-424 in suppression of cell viability, invasive properties, and epithelial-mesenchymal transition (EMT) is mediated through targeting DCLK1. In fact, DCLK1 could partially reverse function of miR-424 in neuroblastoma cells [9].

In MG-63 and SaOS2 osteosarcoma cells, expression of miR-424-5p has been increased upon treatment with melatonin leading to inhibition of VEGFA. Moreover, melatonin could suppress neoangiogenesis, affecting proliferation and migration of neighboring endothelial cells as well as release of angiogenic growth factors. It has also induced morphological changes in blood vessels, and. Taken together, melatonin has a tumor suppressive role through influencing miR-424-5p/VEGFA axis [10].

In silico studies in glioblastoma have predicted a tumor suppressor role for miR-424. This miRNA has also been predicted to target several genes from the ERBB signaling pathway that are activated in the majority of glioblastoma patients. Cell line studies have also confirmed the impact of miR-424 overexpression in suppression of proliferation and migratory potential of glioblastoma cells. Moreover, miR-424 has been shown to promote apoptosis and induce cell-cycle arrest in glioblastoma cells. As predicted by in silico assays, miR-424 could decrease expressions of KRAS, RAF1, MAP2K1, EGFR, PDGFRA, AKT1, and mTOR. Direct interactions between miR-424-5p and RAF1 and AKT1 oncogenes has been verified by dual-luciferase reporter assay [11].

Contrary to glioblastoma cells, expression of miR-424-5p has been reported to be increased in colorectal cancer cell lines. miR-424-5p can promote proliferation and metastatic-related phenotypes through directly binding with SCN4B [12]. In laryngeal squamous cell carcinoma cells, up-regulation of miR-424-5p has enhanced proliferation, migratory aptitude, invasiveness, and adhesion of cancer cells with an important effect on cell cycle progression. In addition, CADM1 has been identified a direct target of miR-424-5p in these cells [13].

An experiment in lung cancer cells has shown that both miR-424-3p and miR-424-5p can prevent proliferation, migration, and invasiveness of these cells. In addition, miR-424-3p but not miR-424-5p could enhance chemosensitivity of lung cancer cells via influencing expression of YAP1 [14].

Different types of Small nucleolar RNA host gene (SNHG) family members have been shown to regulate expression levels of miR-424 in multiple cancers. SNHG family belongs to lncRNAs and have oncogenic roles in the malignancies [15]. For evaluation of their relation to miR-424, following studies have been conducted: In osteosarcoma cell lines Saos-2, MG63, HOS and U2OS, SNHG1 acts as a molecular sponge for miR-424-5p. After knocking down SNHG1, expression levels of miR-424-5p rises and this miRNA can target 3′-UTR of FGF2, resulting in reduced proliferation, migration and invasion [16]. The same molecular mechanism also applies for cervical cancer cells, but the difference is that sponging molecule is SNHG12 in this case and there is no FGF2 targeting [17]. Finally, in T98G and LN229 glioma cells, treatment with Ropivacaine causes SNHG16 levels to drop, and subsequent up-regulation of miR-424-5p happens, which not only suppresses proliferation and migration, but also induces apoptosis in glioma cells [18].

E2F family of TFs (transcription factors) are TFs that were firstly studied in 1987 [19]. This family can either be activators or suppressors of transcription [20]. Interestingly, E2Fs are dysregulated in a variety of cancers and it has been demonstrated that they can be regulated by miR-424. For instance, in hepatocellular carcinoma, forced expression of miR-424-5p and miR-424 is followed by downregulation of E2F7 and E2F3, respectively [21, 22]. Downregulation of these two TFs is favorable and contributes to normal-like cell properties. In cases of endometrial carcinoma, up-regulation of miR-424 diminishes E2F6 and E2F7 levels in HEC-1A, Ishikawa and HEC-1B cells, which in turn inhibits migration, invasion, and colony formation of cells [23, 24]. Lastly, in non-small cell lung cancer cell lines A549 and H460, elevation of miR-424 directly targets expression of E2F6, and consequently reduced proliferation occurs [25].

X-inactive specific transcript (XIST) belong to lncRNAs, and is famously known as X chromosome inactivator in females [26]. It is of great importance to know that this lncRNA can act as a molecular sponge for miR-424-5p in two types of cancer: firstly, in pituitary adenoma cell lines, depletion of XIST is followed by up-regulation of miR-424-5p, and as expected, reduced proliferation, migration and invasion occurs because of bFGF targeting by miR-424-5p [27]. A more detailed mechanism of XIST is demonstrated in hepatocellular carcinoma, in which inhibiting XIST expression contributes to overexpression of miR-424-5p. This miRNA degrades OGT and suppresses RAF1 glycosylation, resulting in favorable normal features in HCC cell lines [28].

Table 1 summarizes the role of miR-424 in different cancer cell lines.

**Table 1:** Role of miR-424 in cancer cell lines (∆: knockdown or deletion, EMT: Epithelial mesenchymal transition, TRAIL: Tumor necrosis factor-related apoptosis-inducing ligand, 5-FU: 5- Fluorouracil, DDP: cisplatin)

Tumor type	Targets/Regulators and signaling pathways	Cell line	Function	References
Neuroblastoma	DCLK1	SK-N-SH and Be2C	↑ miR-424 → ↓ DCLK1: ↓ invasion ↓ EMT process	[9]
Oral squamous cell carcinoma	Circ_0004872	SCC‐6, HN4, SCC‐9, CAL‐27 and SCC‐4	↑ circ_0004872 (which sponges miR-424-5p): ↓ proliferation ↓ invasion ↓ glycolysis ↑ apoptosis	[29]
	CircGDI2/SCAI	CAL-27 and SCC-15	↑ CircGDI2 (which sponges miR-424-5p) → ↑ SCAI: ↓ proliferation ↓ invasion ↓ migration ↓ glycolysis ↑ apoptosis	[30]
Osteosarcoma	miR-424-5p/ VEGFA axis	SaOS2 and MG63	Treatment with melatonin: ↑ miR-424-5p → ↓ VEGFA: ↓ angiogenesis	[10]
Glioblastoma	ERBB pathway: KRAS, RAF1, MAP2K1, EGFR, PDGFRA, AKT1 and mTOR	U-251 and U-87	↑ miR-424-5p (which targets RAF1 & AKT1 and ERRB pathway related genes): ↓ proliferation ↓ migration ↑ apoptosis	[11]
Colorectal cancer	SCN4B	FHC and CRC cell lines (HCT8, HT29, HCT116, SW480, and SW620)	↑ miR-424-5p → ↓ SCN4B: ↑ proliferation ↑ metastasis	[12]
	Src/focal adhesion kinase signaling mediated EMT	5-fluorouracil-resistant HT-29	↑ miR-424-5p → ↓ Src/focal mediated EMT: ↓ resistance to 5-FU	[31]
	AKT3 and PSAT1	HCT116 and RKO	↑ miR-424 → ↓ AKT3 & PSAT1: ↓ proliferation	[32]
	TGFBR3	Lovo	↓ miR-424-5p → ↑ TGFBR3: ↓ proliferation ↑ apoptosis ↓ invasion ↓ migration	[33]
	circTBL1XR1/Smad7	LoVo, SW620, IEC-6, HCT 116 and SW480	∆ circTBL1XR1 (which sponges miR-424) → ↑ miR-424 → ↓ Smad7: ↓ proliferation ↓ invasion ↓ migration	[34]
	-	SW480	∆ miR-424-5p: ↓ proliferation ↓ invasion ↓ migration ↓ colony formation	[35]
Colorectal cancer continued	FENDRR	HCT116, SNU-C2B, NCI-H498 and HCT-15	↑ FENDRR (which sponges miR-424-5p): ↓ proliferation ↓ invasion ↓ migration	[36]
Laryngeal squamous cell carcinoma	CADM1	FD-LSC-1 and TU-177	↑ miR-424-5p → ↓ ADM1: ↑ proliferation ↑ migration ↑ invasion	[13]
Osteosarcoma	CDC25A/CCNA2 /CCNE1	U2OS and HAL	↑ miR-424 → ↓ CCNA2: ↓ proliferation ↓ migration ↓ cell cycle arrest	[37]
	SNHG1/FGF2	Saos-2, MG63, HOS and U2OS	∆ SNHG1 (which targets miR-424-5p) → ↑ miR-424-5p → ↓ FGF2: ↓ proliferation ↓ migration ↓ invasion	[16]
	circ‐LARP4	MG63	↑ circ‐LARP4 (which sponges miR-424): ↓ viability ↑ sensitivity to cisplatin and doxorubicin	[38]
	Fatty acid synthase (FASN)	U2OS	↑ miR-424 → ↓ FASN: ↓ migration ↓ invasion	[39]
	LINC01116/ HMGA2	MG-63	∆ LINC0116 (which silences miR-424-5p via EZH2) → ↑ miR-424-5p → ↓ HMGA2: ↓ viability ↓ migration ↓ invasion ↓ EMT process	[40]
Cutaneous squamous cell carcinoma	LINC00641	A431	↑ LINC00641 → ↓ miR-424 ↓ proliferation ↓ migration ↓ invasion	[41]
Cutaneous malignant melanoma	TINCR/LATS1 axis	M14, A375 and MV3	↑ TINCR (which sponges miR-424-5p) → ↑ LATS1: ↓ proliferation ↑ apoptosis ↓ invasion	[42]
Triple negative breast cancer	PD-L1	MDA-MB-231	↑ miR-424-5p → ↓ PD-L1: ↑ apoptosis	[43]
Breast cancer	PTEN/PI3K/ AKT/mTOR axis | PD-L1	MDA-MB-231	Treatment with Taxol + miR-424-5p → ↓ PTEN/PI3K/ AKT/mTOR axis and PD-L1: ↓ proliferation ↑ apoptosis ↓ colony formation ↑ cell cycle arrest	[44]
	Ginsenoside Rg3/ ATXN8OS/ EYA1, CHRM3, and DACH1 axis	MCF-10A, MCF-7, and MDA-MB-231	Treatment with Rg3 → ↓ ATXN8OS (which sponges miR-424-5p) → ↑ miR-424-5p → ↓ EYA1, CHRM3, DACH1: ↓ proliferation ↑ apoptosis	[45]
	CDK1	MDA-MB-231 and HCC1937	↑ miR-424-5p → ↓ CDK1: ↓ proliferation ↓ colony formation	[46]
	LINC00473/CCNE1	-	∆ LINC00473 (which sponges miR-424-5p) → ↑ miR-424-5p → ↓ CCNE1: ↓ proliferation ↓ migration ↓ invasion ↓ EMT process	[47]
	Bax and Beclin-1 Bcl-2 and c-Myc STAT-3 and Oct-3	MCF-7	↑ miR-424-5p (in combination with miR-142-3p) → ↑Bax and Beclin-1 + ↓ Bcl-2 and c-Myc + ↓ STAT-3 and Oct-3: ↓ proliferation ↑ cell cycle arrest	[48]
	DCLK1	DU4475, HCC1806 and MDA-MB-468	↑ miR-424-5p → ↓ DCLK1: ↓ proliferation ↓ motility	[49]
Renal cancer	CDC2/WEE1	786-O	↑ miR-424 → ↓ WEE1 → ↑ CDC2: ↓ proliferation ↑ apoptosis	[50]
Tongue squamous cell carcinoma	TGFBR3	CAL-27	↑ miR-424 → ↓ TGFBR3: ↑ proliferation ↑ migration ↑ EMT	[51]
Gastric cancer	ABCC2	SGC-7901 and SGC-7901/DDP	↓ miR-424-3p: ↑ resistance to DDP in SGC-7901 ↑ miR-424-3p: ↓ proliferation in SGC-7901/DDP	[52]
	Smad3/TGF-β signaling pathway	MGC803, BGC823, SGC7901, AGS and HGC27	↑ miR-424-5p → ↓ Smad3 → ↓ TGF-β: ↑ proliferation	[53]
	MBNL1-AS1/Smad7	AGS, MGC803, BGC-823, SGC-7901, HGC-27	↑ MBNL1-AS1 → ↓ miR-424-5p → ↑ Smad7: ↓ proliferation ↓ migration ↓ invasion ↑ apoptosis	[54]
	Circular RNA_LARP4/LATS1	SGC-7901, MKN-45, MKN-28, HGC-27, MGC-803, AGS and BGC-823	↑ LARP4 (which sponges miR-424-5p) → ↑ LATS1: ↓ proliferation ↓ invasion	[55]
Esophageal squamous cell carcinoma	SIRT4	HEEC, EC9706, Eca-109, KYSE-150 and TE-1	↑ miR-424-5p → ↓ SIRT4: ↑ proliferation	[56]
	Smad7	EC9706, Eca109, EC-1	↑ miR-424-5p → ↓ Smad7: ↓ proliferation ↓ invasion ↓ EMT process	[57]
Acute myeloid leukemia	PLAG1	HL-60, NB4, HL-60/ADM, K562	↑ miR-424 → ↓ PLAG1: ↑ sensitivity to TRAIL	[58]
Prostate cancer	ESE3/EHF /COP1/STAT3	DU145	↓ ESE/EHF → ↑ miR-424-5p → ↓ COP1 (which degrades STAT3) → ↑ STAT3: ↑ proliferation ↑ migration	[59]
Infantile hemangioma	VEGFA	XPTS-1	Treatment with propranolol: ↑ miR-424 → ↓ VEGFA: ↓ viability ↓ invasion ↑ apoptosis	[60]
Pituitary adenoma	JAG1	GH3	↑ miR-424-3p → ↓ JAG1: ↓ proliferation	[61]
	XIST/bFGF axis		∆ XIST (which sponges miR-424-5p) → ↑ miR-424-5p → ↓ bFGF: ↓ proliferation ↓ migration ↓ invasion ↑ apoptosis	[27]
Cervical cancer	KDM5A/Suz12	CaSki	Human papillomavirus type 16 E7 induction: ↑ KDM5A (which binds to promoter region of miR-424-5p and inhibits it) → ↑ Suz12: ↑ proliferation ↑ invasion	[62]
	SNHG12	C33A, ME-180, CaSki, HeLa and SiHa	∆ SNHG12 (which sponges miR-424-5p) → ↑ miR-424-5p: ↓ proliferation ↓ migration ↓ invasion	[17]
Head and Neck Squamous Cell Carcinoma	LAMC1/ Wnt/β-catenin signaling pathway	HUVEC	↑ miR-424-5p → ↓ LACM1 → ↓ Wnt/β-catenin signaling pathway: ↓ angiogenesis ↓ migration	[63]
Hepatocellular carcinoma	E2F7	HB-8064, HB-8065, CRL2235, CRL-2237 and THLE-3	↑ miR-424-5p → ↓ E2F7: ↓ proliferation ↑ cell cycle arrest	[21]
	KIF2A	Huh7 and HepG2	↑ miR-424-5p (which is epigenetically suppressed) → ↓ KIF2A: ↓ EMT process ↓ proliferation ↑ apoptosis	[64]
	XIST/OGT/RAF1	MHCC97L, MHCC97H, SNU-398, SMMC7221, and Huh7	↓ XIST (which sponges miR-424-5p) → ↑ miR-424-5p → ↓ OGT → ↑ RAF1: ↓ EMT process ↓ proliferation	[28]
	ATG14	HepG2, SMMC-7721, Huh-7, MHCC97H and HCCLM3	↑ miR-424-5p → ↓ ATG14: ↓ proliferation ↑ apoptosis ↓ autophagy	[65]
	-	SMMC-7721, Huh-7, HepG2, Bel-7402, and SK-HEP-1	↑ miR-424: ↓ proliferation ↓ migration ↓ invasion	[66]
	CircCBFB/ATG14	Huh-7 and HCCLM3	↑ CircCBFB (which sponges miR-424-5p) → ↑ ATG14: ↑ proliferation ↑ autophagy	[67]
	CDKN2B-AS1	Huh7, Hep3B and Sk-Hep1	↓ CDKN2B-AS1 (which sponges miR-424-5p) → ↑ miR-424-5p: ↓ proliferation ↓ migration ↓ invasion ↓ EMT process	[68]
Hepatocellular carcinoma continued	TRIM29	MHCC-97H, HepG2, SMMC-7721, and Huh-7	↑ miR-424-5p → ↓ TRIM29: ↓ proliferation ↓ migration ↓ invasion ↓ colony formation	[69]
	Akt3/E2F3 axis	SMMC7721, HepG2, HUH7, MHCC97-L, MHCC97-H and HCCLM3	↑ miR-424 → ↓ Akt3/E2F3: ↓ proliferation ↓ colony formation	[22]
	LINC00922/ARK5 axis	SNU-182 and SK-Hep1	↑ LIN C00922 → ↓ miR-424-5p → ↑ ARK5: ↑ viability ↑ migration ↑ invasion ↑ EMT process	[70]
	DLX6-AS1/WEE1	SK‐HEP‐1 and Hep3B	↑ DLX6-AS1 (which sponges miR-424-5p) → ↑ WEE1: ↑ proliferation ↑migration ↑ invasion	[71]
Cholangiocarcinoma	LINC00665/BCL9L	HuCCT1-Gem and SNU-245-Gem	∆ LINC00665 (which sponges miR-424-5p) → ↑ miR-424-5p → ↓ BCL9L: ↑ apoptosis ↓ EMT process ↓ resistance to gemcitabine	[72]
Intrahepatic cholangiocarcinoma	ARK5	CLP-1, RBE and HuCCT-1	↑ miR-424-5p → ↓ ARK5: ↓ migration ↓ invasion	[73]
Bladder cancer	DNMT1/ EGFR signaling	HT1197 and HT1376	↑ DNMT1 (which suppresses miR-424 expression): ↑ EGFR signaling: ↑ proliferation ↑ migration ↑ EMT process	[74]
	LINC00355/ HMGA2	T24, HT-1197, SW780, HT-1376, UM-UC-3, TCCSUP, KU1919, and VMCUB1	↑ LINC00355 (which sponges miR-424-5p) → ↑ HMGA2: ↑ migration ↑ invasion ↑ EMT process	[75]
Endometrial carcinoma	MMSET	Ishikawa and HEC-1	↑ miR424 → ↓ MMSET: ↓ invasion ↓ sphere formation	[76]
	IGF-1R	HEC‐1A, HEC‐1B, AN3CA, and Ishikawa	↑ miR-424 → ↓ IGF‐1R: ↓ viability ↓ proliferation ↓ EMT process	[77]
	E2F6	HEC-1A, Ishikawa	↑ miR-424-3p → ↓ E2F6: ↓ migration ↓ invasion ↓ EMT process	[23]
	E2F7	Ishikawa and HEC-1B	↑ miR-424 → ↓ E2F7: ↓ proliferation ↓ colony formation ↑ apoptosis	[24]
	SPTBN2 /PI3K/AKT	AN3C and Ishikawa	↑ miR-424-5p → ↓ SPTBN2 → ↓ PI3K/AKT: ↓ proliferation ↓ colony formation	[78]
Ovarian cancer/epithelial ovarian cancer (EOC)	LGALS3	SK-OV-3 and TOV-21G	↑ miR-424-3p → ↓ LGALS3: ↑ sensitivity to cisplatin	[79]
	NANOG/WEE1	SKOV3, OVCAR3, OVCAR5, and OVCAR8	↑ miR-424 (which is suppressed by NANOG) → ↓ WEE1: ↓ proliferation ↓ migration ↓ colony formation ↑ sensitivity to carboplatin	[80]
	MYB	SKOV-3, HO8910 and A2780	↑ miR-424-5p → ↓ MYB: ↓ proliferation ↓ migration ↓ invasion	[81]
	CCNE1	SKOV3, HO8910 and A2780	↑ miR-424-5p → ↓ CCNE1: ↓ proliferation ↑ cell cycle arrest	[82]
	ACSL4	HO8910 and SKOV3	↑ miR-424-5p → ↓ ACSL4: ↓ ferroptosis	[83]
Ovarian clear cell carcinoma	DCLK1	ES-2 and RMG-1	↑ miR-424 → ↓ DCLK1: ↓ migration ↓ invasion ↓ EMT process	[84]
Pancreatic ductal adenocarcinoma (PDAC)	SOCS6	PANC-1, AsPC-1, BxPC-3 and MIAPaCa-2	↓ miR-424-5p → ↑ SOCS6: ↓ proliferation ↓ migration ↓ invasion ↑ apoptosis	[85]
Glioma	CCAT2/VEGFA	A172, U251 and HCMEC/D3	↑ miR-424 (which is sponged by CCAT2) → ↓ VEGFA: ↓ Angiogenesis	[86]
	GAS5/PRC2	LN229, A172, U373, SHG44 and NHA	↑ GAS5 (which interacts with EZH2 in PRC2) → ↓ methylation of miR-424 promoter → ↑ miR-424: ↑ apoptosis ↓ proliferation ↓ migration ↓ invasion	[87]
	KIF23	A172, SHG-44, T98, LN18, and LN229	↑ miR-424 → ↓ KIF23: ↓ EMT process	[88]
	FAM87A/PPM1H PI3K/Akt Signaling Pathway	T98G, A172, SNB19 and U251	↑ FAM87A (which sponges miR-424-5p) → ↑ PPM1H → ↓ PI3K/Akt Signaling Pathway: ↓ viability ↓ migration ↓ invasion	[89]
	SNHG16	T98G and LN229	Treatment with Ropivacaine: ↓ SNHG16 (which targets miR-424-5p) → ↑ miR-424-5p: ↑ apoptosis ↓ proliferation ↓ migration ↓ invasion	[18]
Nasopharyngeal carcinoma	AKT3	NPC-1	↑ miR-424-5p → ↓ AKT3: ↓ proliferation ↓ migration ↓ invasion	[90]
Non-small cell lung cancer	YAP1	H827, H2172, H441, A549, H1975, and PC14	∆ miR-424-3p (which targets YAP1): ↑ proliferation ↑ migration ↑ invasion ↑ resistance to Paclitaxel	[14]
	PTEN/PI3K/Akt pathway	A549 and H460	Treatment with baicalein: ↓ miR‐424‐3p → ↑ PTEN → ↓ PI3K/AKT: ↑ cisplatin sensitivity ↓ cell survival	[91]
	E2F6	A549 and H460	↑ miR-424 → ↓ E2F6: ↓ proliferation ↓ invasion	[25]
	–	H596 and SW900	↓ miR-424: ↓ proliferation ↑ cell cycle arrest	[92]

## Animal models

Animal models of different types of cancers, including mammary tumors, aggressive osteosarcoma, gastric cancer, esophageal squamous cell carcinoma, thyroid cancer and glioma have been established to assess the impact miR-424 dysregulation on the tumor burden (Table 2). In gastric cancer xenograft models, the results of two studies are contradictory. While up-regulation of miR-424-3p has led to reduction of tumor growth and metastasis in one study [52], another study has reported that over-expression of mir-424-5p has the opposite effect [53]. In esophageal squamous cell carcinoma, both conducted studies have confirmed an oncogenic role for this miR-424 [56, 93]. The results of other studies in xenograft models are shown in Table 2.

**Table 2:** Impact of miR-424 in carcinogenesis in vivo (∆ knockdown or deletion, SCID: severe combined immunodeficiency, SPF: specific pathogen-free)

Tumor type	Animal models	Results	References
Osteosarcoma	SCID mice	↑miR-424: ↓tumor growth	[37]
Gastric cancer	BALB/c nude mice	↑ miR-424-3p: ↓ tumor growth ↓ metastasis	[52]
	BALB/c nude mice	↑ mir-424-5p: ↑ tumor size	[53]
Esophageal squamous cell carcinoma	BALB/c-nude mice	∆ miR-424-5p: ↓tumor growth	[56]
	BALB/c nude mice	↑ miR-424: tumorigenesis	[93]
Thyroid cancer	BALB/c nude mice	∆ miR-424-5p: ↓ metastasis | ↑ miR-424-5p: ↑ Lung metastasis	[94]
Ovarian cancer	Nude mice	↑ miR-424-5p ↓ tumor growth ↓ angiogenesis	[81]
Hepatocellular carcinoma	Nude mice	↑ miR-424-5p: ↓ tumor growth	[64]
	SPF BALB/c nude mice	↓ XIST: ↑ miR-424-5p: ↓ tumor growth	[28]
	BALB/c nude mice	↑ miR-424-5p: ↓ tumor growth	[69]
	Nude mice	↑ miR-424-5p: ↓ tumor growth	[66]
	Nude mice	↑ miR-424-5p: ↓ tumor growth	[22]
Colorectal cancer	BALB/c nude mice	↓ miR-424-5p ↓ tumor growth	[33]
Glioma	BALB/c nude mice	↑ miR-424: ↓ tumor growth	[88]

## Assays in clinical samples

Expression of miR-424 has been evaluated in a variety of malignant tissues. Experiments in ovarian cancer, cervical cancer, hepatocellular carcinoma, neuroblastoma, breast cancer, osteosarcoma, intrahepatic cholangiocarcinoma, prostate cancer, endometrioid endometrial cancer, non-small cell lung cancer, hemangioma and gastric cancer have reported down-regulation of miR-424. On the other hand, this miRNA has been found to be up-regulated in melanoma, laryngeal and esophageal squamous cell carcinomas, glioma, multiple myeloma and thyroid cancer. In colorectal cancer, both patterns have been reported (Table 3). In neuroblastoma tissues, down-regulation of miR-424 has been found to be accompanied by up-regulation of its target gene DCLK1 [9]. On the other hand, miR-424-5p has been found to be upregulated in laryngeal squamous cell carcinoma samples versus adjacent normal margin tissues. Over-expression of miR-424-5p has been associated with poor differentiation, advanced tumor stages and cervical lymph node involvement. In silico analyses have shown that target genes of this miRNA are principally enriched in cell cycle, cell division, and negative regulation of cell migration [13]. In patients with cervical cancer, down-regulation of miR-424 has been reported to be associated with low level of differentiation of cancer cells, advanced clinical stages and metastasis to lymph nodes [95]. Furthermore, in patients with non-small cell lung cancer, low levels of miR-424-3p have been associated with disease progression and overall prognosis [14].

**Table 3:** Dysregulation of miR-424 in clinical specimens (O-S: overall survival, DFS: disease-free survival, ANT: adjacent normal tissue, CFFS: Clinical failure-free survival, TCGA: the cancer genome atlas, AFP: Alpha-fetoprotein, HBV: hepatitis B virus, HPV: human papilloma virus, RFS: recurrence-free survival)

Tumor type	Samples	microRNA Type	Expression (tumor vs normal)	Kaplan–Meier analysis (impact of miR-424)	Association of miR-424 expression with clinicopathologic characteristics	References
Ovarian cancer (OC)/ epithelial ovarian cancer (EOC)	38 OC + paired ANT	5p	Downregulated	–	–	[83]
	31 EOC + paired ANT	miR-424	Downregulated	Lower O-S and DFS	–	[96]
	85 OC + 43 paired ANT	5p	Downregulated	–	–	[81]
	83 EOC + 19 paired ANT	5p	Downregulated	Shorter O-S and RFS	Associated with poor differentiation, advanced FIGO stage, residual tumor size and lymph node metastasis	[82]
Ovarian clear cell carcinoma (OCCC)	30 OCCC + paired ANT	miR-424	Downregulated	–	–	[84]
Cervical cancer (CC)	30 CC + paired ANT	miR-424	Downregulated	Poor O-S	–	[99]
	147 CC + 74 normal tissues	miR-424	Downregulated	Poor O-S	Associated with tumor differentiation, advanced clinical stages and lymph node metastases	[95]
	42 HPV16 positive + 17 HPV16 negative + 13 normal tissues	5p	Downregulated (especially in HPV16 positive)	–	–	[62]
Hepatocellular carcinoma (HCC)	156 HCC + paired ANT	5p	Downregulated	–	–	[100]
	60 HCC + paired ANT	miR-424	Downregulated	Poor O-S	Associated with advanced clinical stage	[101]
	127 HCC + paired ANT	miR-424	Downregulated	Poor O-S	Associated with Lymph node metastasis, vascular invasion, and clinical stage	[102]
	30 HCC + paired ANT	5p	Downregulated	–	–	[64]
	80 HCC + paired ANT	5p	Downregulated	–	–	[28]
	36 HCC + paired ANT	5p	Downregulated	Poor survival rate	Associated with tumor size, HBV infection, AFP content, and TNM	[65]
	90 HCC + paired ANT	5p	Downregulated	Poor O-S	Associated with AFP, TNM stage and intrahepatic metastasis	[69]
	121 HCC + paired ANT	miR-424	Downregulated	–	Associated with recurrence	[66]
	96 HCC + paired ANT	miR-424	Downregulated	Poor O-S	Associated with tumor size, Tumor nodule number, TNM stage and BCLC stage	[22]
	50 HCC + paired ANT	5p	Downregulated	–	–	[103[
	40 HCC + paired ANT	5p	Downregulated	–	–	[70]
Melanoma	Serum and tissue of melanoma patients	miR-424	Upregulated	Lower O-S and DFS	Associated with tumor thickness, metastasis and tumor stage and ulceration	[104]
Cutaneous malignant melanoma (CMM)	60 CMM + paired ANT	5p	Downregulated	–	Associated with advanced TNM stage	[42]
Neuroblastoma	49 neuroblastoma tissues + paired ANT	miR-424	Downregulated	–	–	[9]
Breast cancer	TCGA database	miR-424	Downregulated	Poor O-S	Associated with high-grade, larger tumor size, triple-negative status, stronger cell proliferation, and GGI signature	[105]
	17 BC + paired ANT	5p	Downregulated	Poor O-S	–	[46]
	84 BC + 20 paired ANT	5p	Downregulated	–	Associated with clinical stage, larger tumor size, lymph node metastasis and distant metastasis	[49]
Colorectal cancer (CRC)	GSE108153 (21 CRC + paired ANT) + TCGA database	5p	Upregulated	–	–	[12]
	24 CRC + paired ANT	miR-424	Downregulated	–	–	[106]
	65 CRC + paired ANT	5p	Upregulated	–	Associated with tumor differentiation, tumor infiltration depth, TNM stage, vascular invasion, lymph node metastasis and distant metastasis	[33]
	59 CRC + paired ANT	5p	Upregulated	–	Associated with Dukes’ stage, depth of invasion and pathological type	[35]
	20 CRC + paired ANT	5p	Upregulated	–	–	[36]
Laryngeal squamous cell carcinoma (LSCC)	106 LSCC + paired ANT	5p	Upregulated	Poor O-S	Associated with advanced T stage and lymph node metastasis	[13]
Nasopharyngeal carcinoma (NPC)	40 NPC + 26 healthy controls skin (obtained from plastic surgery)	5p	Downregulated	–	Associated with TNM stage and lymph node metastasis	[90]
Oral squamous cell carcinoma (OSCC)	Saliva of 43 OSCC + 44 healthy controls	3p	Downregulated	–	–	[107]
	60 OSCC + paired ANT	5p	Upregulated	–	–	[29]
	Blood sample of 15 OSCC + 15 healthy controls	5p	Upregulated	–	–	[97]
	30 OSCC + paired ANT	5p	Upregulated	–	–	[30]
Osteosarcoma (OS)	Plasma of 20 OS + 15 healthy controls	miR-424	Downregulated	–	–	[37]
	61 OS + paired ANT	5p	Downregulated	–	–	[16]
Intrahepatic cholangiocarcinoma (ICC)	19 ICC + paired ANT	5p	Downregulated	–	–	[73]
Prostate cancer (PCa)	535 PCa tissues	3p	Downregulated	Poor CFFS	Associated with aggressiveness of disease	[108]
	48 PCa + 21 healthy controls tissue	5p	Upregulated	–	–	[59]
Endometrioid Endometrial Cancer (EEC)/Endometrial carcinoma (EC)	24 EEC + paired ANT	5p	Downregulated	–	–	[78]
	50 EC + 10 fibroid samples as controls	miR-424	Downregulated	–	–	[77]
	32 EC + paired ANT	3p	Downregulated	–	Associated with clinical stage and lymph node metastasis	[23]
	11 EC + paired ANT	miR-424	Downregulated	–	–	[24]
Non-small cell lung cancer (NSCLC)	90 NSCLC + paired ANT	5p	Downregulated	Poor O-S	Associated with clinical stage, lymph node metastasis, differentiation degree, and tumor volume	[14]
	233 NSCLC + paired ANT	miR-424	Upregulated in advanced stage (no difference with normal tissues)	Poor O-S	Associated with advanced clinical stage, TNM stage and lymph node metastasis	[92]
Glioma	76 glioma tissues + paired ANT	5p	Upregulated	Poor O-S	–	[89]
	54 glioma tissues + 12 healthy controls tissues (obtained from traumatic brain injury)	miR-424	Downregulated	Poor O-S	Associated with WHO grade and KPS	[88]
Esophageal squamous cell carcinoma cell (ESCC)	30 ESCC + 10 healthy controls	miR-424	Upregulated	Poor O-S	–	[93]
	32 ESCC + paired ANT	5p	Downregulated	–	Associate with differentiation and lymph node metastasis	[57]
Hemangioma (HM)	7 Senile HM + 3 venous malformations + 4 angiosarcoma + 4 venous lakes + 3 infantile HM	miR-424	Downregulated	–	Associate with Abnormal Angiogenesis	[109]
Infantile hemangioma (IH)	16 IH + 16 normal subcutaneous tissues	miR-424	Downregulated	–	–	[110]
	13 IH + paired ANT	miR-424	Downregulated	–	–	[60]
Gastric cancer (GC)	48 GC + paired ANT	miR-424	Downregulated	Poor O-S	Associated with TNM stage and lymph node metastasis	[98]
	60 GC + paired ANT	5p	Upregulated	–	–	[54]
	TCGA database (387 GC + 41 paired ANT)	5p	Upregulated	Recurrence of GC	–	[55]
Pancreatic ductal adenocarcinoma (PDAC)	24 PDAC + paired ANT	5p	Upregulated	–	–	[85]
Multiple myeloma (MM)	Serum of 81 MM patients + 50 healthy controls	miR-424	Upregulated		Associated with clinical stage	[111]
Thyroid cancer (TC)	TCGA dataset + 10 TC + paired ANT	5p	Upregulated	–	Associated with distant metastasis	[94]

Out of 4 different studies conducted on ovarian cancer/epithelial ovarian cancer patients, two have reported downregulation of miR-424 and other two verified downregulation of miR-424-5p in tumor tissues compared with adjacent normal tissues [81–83, 96].

In Oral squamous cell carcinoma (OSCC), three different studies confirmed up-regulation of miR-424-5p both in OSCC tissues and blood samples [29, 30, 97]. Discordant to mentioned studies, miR-424-3p has shown to be under-expressed in saliva of OSCC patients, and its levels can be used as a diagnostic marker to differentiate OSCC patients and healthy controls, with an AUC value of 0.732, sensitivity of 0.605 and 0.818 specificity.

It is worth mentioning that a great number of research on clinical specimens have been conducted in hepatocellular carcinoma (HCC) patients. In total, after evaluating expression of miR-424 and miR-424-5p in 886 pairs of HCC tissues in 11 independent studies, it is concluded that both of these miRNAs are downregulated in HCC tissues. According to Kaplan–Meier analysis, downregulated levels of miR-424-5p is associated with shorter over-all survival in HCC patients [65]. In addition, diminished levels of this miRNA is associated with alpha-fetoprotein content and HBV infection in HCC [65].

In gastric cancer (GC) patients, miR-424 has been verified to be downregulated [98] but miR-424-5p is upregulated in tumor expression profile [54, 55]. In case of miR-424 downregulation, NNT-AS1 acts as a molecular sponge and inhibits miR-424 and this phenomenon is accompanied by activation of E2F1, a transforming transcription factor [98].

Associations between dysregulation of miR-424 and clinical outcome have been demonstrated in ovarian, cervical, breast, prostate, lung, melanoma, colorectal and other cancers (Table 3).

In patients with colorectal cancer, cancer cells have been shown to release miR-424-5p into peripheral blood in the form of exosomes. Notably circulating exosomal levels of miR-424-5p can separate patients with early stage of colorectal cancer from healthy individuals with area under the ROC curve (AUC) value of 0.82 [12]. In patients with multiple myeloma, serum levels of this miRNA could be used as a diagnostic marker with AUC value of 0.95 [111]. Diagnostic role of miR-424 has also been evaluated in hepatocellular carcinoma, prostate cancer and renal cell carcinoma revealing different AUC values (Table 4).

**Table 4:** Diagnostic value of miR-424 in cancers (BPH: benign prostate hyperplasia)

Tumor type	Samples	Distinguish between	Area under curve	Sensitivity (%)	Specificity (%)	References
Hepatocellular carcinoma (HCC)	Serum of HCC patients and healthy controls	HCC vs healthy controls	0.768	75.0%	72.4%	[112]
Multiple myeloma (MM)	81 MM + 50 healthy controls	MM vs healthy controls	0.952	95.0%	87.2%	[111]
Prostate cancer (PC)	Serum of 36 PCa + 54 BPH	PCa vs BPH	0.671	31.94%	94.87%	[113]
Oral squamous cell carcinoma (OSCC)	Saliva of 43 OSCC + 44 healthy controls	OSCC vs healthy controls	0.732	0.605	0.818	[107]
Renal cell carcinoma (RCC)	Serum of 22 RCC + 16 healthy controls	RCC vs healthy controls	0.7727	81.8%	75.0%	[114]

## Discussion

**Fig. 1:**
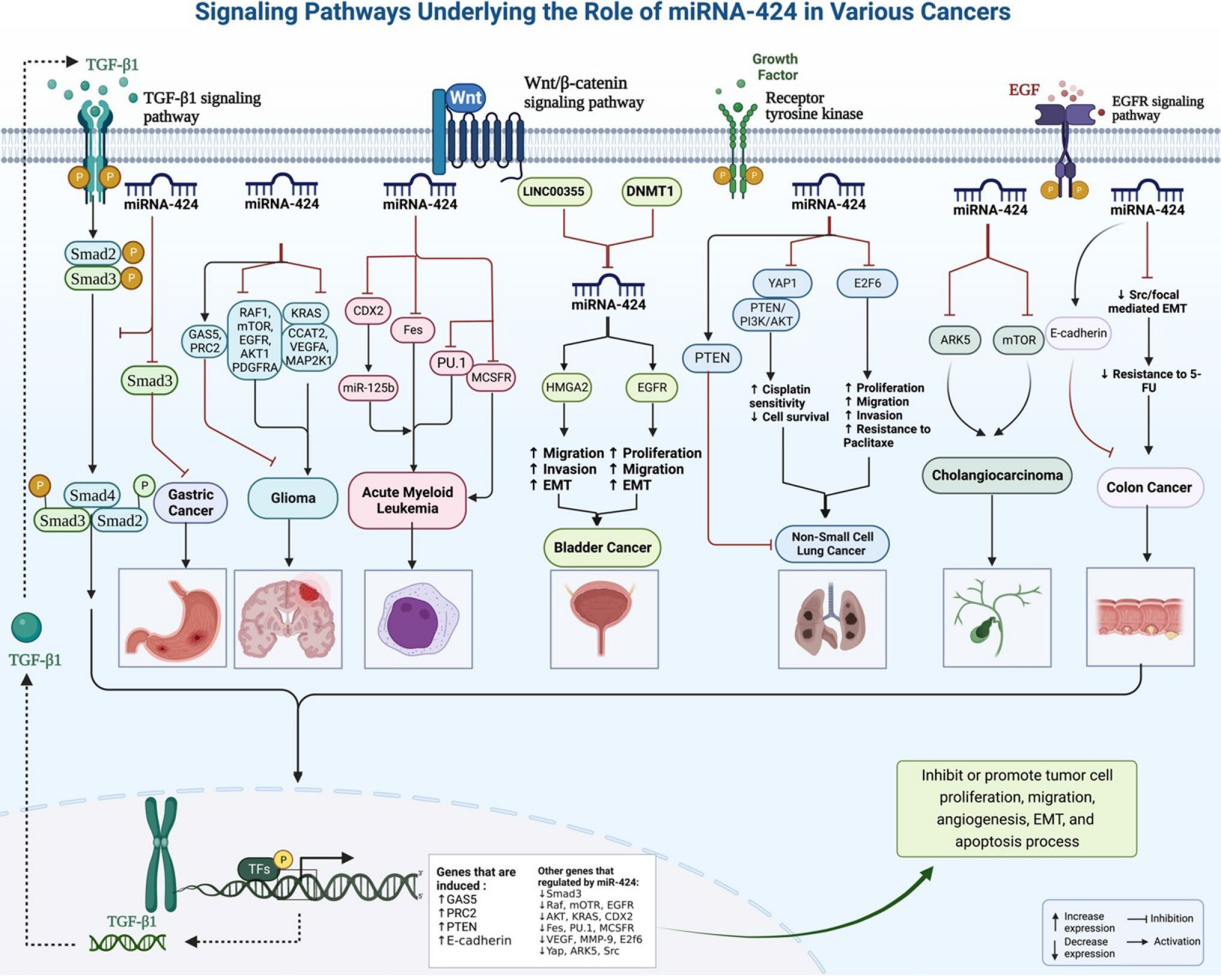
miRNA-424 can either inhibit or stimulate cancer cell growth depending on the context. For instance, overexpression of miR-424 can inhibit glioblastoma cell growth and migration. In addition, miR-424 has been demonstrated to induce cell-cycle arrest and enhance apoptosis in glioblastoma cells. KRAS, RAF1, MAP2K1, EGFR, PDGFRA, AKT1, and mTOR expressions may all be suppressed by miR-424, as suggested by in silico experiments

miR-424 is an example of miRNAs with crucial roles in the carcinogenesis. However, its role in this process may depend on the type of tissue, since in some tissues it makes cancer cells grow and in others it prevents cancer cells from growing. Moreover, it is possible that miR-424-3p and miR-424-5p exert different roles in the process of carcinogenesis in some cases (115) (Fig. 1). This is mostly related with the specific targets of miR-424 in each tissue. The interactions between miR-424 and tissue specific transcription factors might also been involved in the specificity of miR-424 functions in each tissue.

Expression of this miRNA is regulated by methylation status of its promoter and lncRNAs that sponge this miRNA [64]. LINC00641 [115], CCAT2 [116], PVT1 [117], LINC00511 [102] and NNT-AS1 [98] are among lncRNAs that act as molecular sponges for miR-424. The interactions between lncRNAs and miRNAs forms a huge and complex regulation network for regulation of gene expression at transcriptional, post-transcriptional, and post-translational levels [118]. This multilevel regulatory network is involved in the carcinogenesis. miR-424/lncRNA axes might represent potential targets for design of novel therapeutics for cancers. However, the function of these axes should be individually assessed in each type of cancer.

miR-424 has a regulatory role on activity of VEGFA, ERBB, mTOR, TGF-β and PTEN/PI3K/AKT pathways. Since miR-424 can influence expression of VEGFA, it has a pivotal role in the regulation of tumor angiogenesis [119]. Moreover, it has been shown to influence EMT process through inhibiting mTOR phosphorylation [73]. miR-424 can affect cell-cycle transition and cell apoptosis. KRAS, RAF1, MAP2K1, EGFR, PDGFRA, AKT1, and mTOR expressions can also been affected by miR-424 [11]. Therefore, a wide array cancer-related genes, pathways and cellular functions are controlled by this miRNA.

Serum levels of miR-424 can distinguish cancer patients from healthy controls with variable diagnostic power values. Diagnostic role of miR-424 has been evaluated in hepatocellular carcinoma [112], multiple myeloma [120], prostate cancer [121], oral squamous cell carcinoma [107], renal cell carcinoma [50] with the best values being obtained in multiple myeloma. Confirmation of these results in larger sample sizes from different types of cancer can broaden application of this miRNA in non-invasive methods for cancer detection. Since early diagnosis of cancer is the most efficient way to increase survival of patients, these findings have significance in this regard. Moreover, serum levels of miR-424 can be used for follow-up of patients with different types of cancers after conduction of anti-cancer therapies to detect cancer recurrence.

The association between expression levels of miR-424 and clinical outcome of patients further highlights the potential of this miRNA as a predictive biomarker in cancer patients. However, since miR-424 has opposite roles in different cancers [13], the patterns and direction of these associations depends on the role of miR-424 in each type of cancer. This note should also be considered when designing miR-424-targeting strategies in the treatment of cancer. Although there are ongoing clinical trials in phase 1 and 2 regarding their therapeutic application, there is no FDA approved miRNA-based drug in the market [122]. However, patisiran, and givosiran are two FDA approved siRNA-based drugs [123] and because of the similarity in the mechanism of action, we can anticipate miRNA-based drugs in the near future. In the cases of miR-424, the wide range of molecules being affected by this miRNA enhances the efficacy of targeted therapies in the field of cancer. However, this feature also increases the possibility of unwanted side effects.

Since the majority of studies, especially cell line studies are conducted on colorectal cancer (7 studies), osteosarcoma (5 studies), breast cancer (7 studies), gastric cancer (4 studies), hepatocellular carcinoma (11 studies), endometrial carcinoma (5 studies), ovarian cancer (6 studies), glioma (5 studies) and non-small cell lung cancer (4 studies), it might be wise to be more focused on these conditions when considering therapeutic approaches of miR-424.

Finally, miR-424 affects response of cells to 5-flurouracil [31], paclitaxel [14], gemcitabine [72] and cisplatin [52]. Thus, dysregulation of expression of miR-424 might be involved in the chemoresistance phenotype.

In brief, miR-424 is an example of miRNAs with tissue-specific impacts in the carcinogenesis. Experiments in cancer cell lines and animal models of cancer have shown feasibility and efficacy of miR-424-targeting strategies in decreasing invasiveness of cancer cells and tumor burden, respectively. Applicability of these strategies in clinical setting has not been evaluated yet. Future studies are needed to elaborate this aspect.

## Data Availability

The analyzed data sets generated during the study are available from the corresponding author on reasonable request.
